# Risk Factors for Emergency Department Short Time Readmission in Stratified Population

**DOI:** 10.1155/2015/685067

**Published:** 2015-11-17

**Authors:** Ariadna Besga, Borja Ayerdi, Guillermo Alcalde, Alberto Manzano, Pedro Lopetegui, Manuel Graña, Ana González-Pinto

**Affiliations:** ^1^Emergency Department, Álava University Hospital, 01010 Vitoria, Spain; ^2^Biomedical Research Networking Center in Mental Health (CIBERSAM), 10001 Madrid, Spain; ^3^Faculty of Medicine, University of the Basque Country (UPV/EHU), 01010 Vitoria, Spain; ^4^Computational Intelligence Group (GIC), UPV/EHU, 20018 San Sebastián, Spain; ^5^ACPySS, 20018 San Sebastián, Spain; ^6^Management, Álava University Hospital, 01010 Vitoria, Spain; ^7^Department of Psychiatry, Álava University Hospital, 01010 Vitoria, Spain

## Abstract

*Background.* Emergency department (ED) readmissions are considered an indicator of healthcare quality that is particularly relevant in older adults. The primary objective of this study was to identify key factors for predicting patients returning to the ED within 30 days of being discharged.* Methods.* We analysed patients who attended our ED in June 2014, stratified into four groups based on the Kaiser pyramid. We collected data on more than 100 variables per case including demographic and clinical characteristics and drug treatments. We identified the variables with the highest discriminating power to predict ED readmission and constructed classifiers using machine learning methods to provide predictions.* Results.* Classifier performance distinguishing between patients who were and were not readmitted (within 30 days), in terms of average accuracy (AC). The variables with the greatest discriminating power were age, comorbidity, reasons for consultation, social factors, and drug treatments.* Conclusions.* It is possible to predict readmissions in stratified groups with high accuracy and to identify the most important factors influencing the event. Therefore, it will be possible to develop interventions to improve the quality of care provided to ED patients.

## 1. Introduction

Population ageing is one of the most important sociodemographic changes in recent years. It is expected that, in the near future, people over 65 years will be 21.2% of the overall population. This trend will continue having a great impact on the health system, especially in emergency departments (EDs) [1]. The increased healthcare needs for this growing age group represent an unprecedented challenge. In developed countries, older adults already account for 12 to 21% of all ED visits and it is estimated that this will increase by around 34% by 2030 [2].

Compared to younger patients, older patients have increasingly complex medical conditions in terms of their number of illnesses and the characteristics of, thereof, the number of medications they use, geriatric syndromes, their degree of physical or mental disability, and the interplay of social factors influencing their condition [3, 4]. Further, recent studies have shown that adults, and in particular those above 75 years of age, have the highest rates of ED readmission, and longest stays, and require around 50% more ancillary tests including imaging and laboratory studies [5, 6]. However, despite the intense use of resources, these patients often leave the ED unsatisfied and, compared to younger patients, with poorer clinical outcomes and higher rates of misdiagnosis and medication errors [7]. Additionally, once they are discharged from hospital, they have a high risk of adverse outcomes, such as functional worsening, ED readmission, hospitalisation, death, and institutionalisation [8].

EDs are designed to care for acutely ill patients with single health problems. The need for triage and rapid intervention makes it difficult to provide proper care to patients with complex characteristics and, on the other hand, such patients slow down the functioning of EDs, sometimes even overloading them [8]. A systematic framework for handling older patients will help to make the ED process safer and more efficient.

The population covered by the public health system in the Basque Country (Spain) is stratified according to the level of complexity of diagnosis and treatment, with the objective of ensuring that the specific needs of older adults are met at the different levels of care provision [1]. According to the Kaiser Permanente pyramid, the population is stratified into three levels. At its base (level 1), we find healthy members of the population; the second level includes patients with prominence of specific organ disease (heart failure (HF), chronic obstructive pulmonary disease (COPD), and diabetes mellitus (DM)), and the third level includes the most complex patients having high multimorbidity, who are candidates for comprehensive care plans. In these plans, EDs are at the interface between the hospital and primary care, representing a key link for identifying prevention and follow-up strategies [9].

If we are able to identify the most relevant factors for the prediction of readmission in patients in levels 2 and 3, these factors may be taken into account for the design of improved operation of the ED. They can also be considered as indicators of the quality of care provided. Finally, prediction of readmission will allow better planning of the resources for improvement in terms of clinical effectiveness and efficiency.

## 2. Methods

We analysed patients stratified at levels 2 and 3 who attended the ED of the Araba University Hospital (AUH) during June 2014, divided into four groups: patients identified as requiring case management (CM), these corresponding to individuals in level 3 (*n* = 99), and patients with COPD (*n* = 81), with HF (*n* = 85), or with DM (*n* = 126).

The control variable was the time between readmissions, this being used to divide the total sample into two classes: readmitted patients, those returning to the ED within 30 days after being discharged, and nonreadmitted patients. The classification problem consisted of predicting patient class from clinical and sociodemographic data.

The study variables include sociodemographic data, personal medical history, reasons for consultation, and regular medications. The full list of variables is given in the Supplementary Material available online at http://dx.doi.org/10.1155/2015/685067. This study was approved by the Ethics Committee of the hospital. The anonymous data has been published in the research group web page (http://www.ehu.eus/ccwintco/index.php?title=Dato-emergencias). The description of the variables measured for each patient is given in the Supplementary Material.

### 2.1. Statistical Analysis and Data Processing

In this section, we describe the analysis performed over the data. We have tested the statistical significance of differences between populations, the predictive power of the variables, akin to their importance, and the expected predictive performance that may be achieved using the selected variables. With multivariate analysis, it is possible to analyse many variables at the same time, taking into account their interactions and correlations, both for the classification of a population into groups using classification algorithms and for predicting control variables using regression algorithms. In this study, we used classifiers based on support vector machines (SVM), which have been accepted as standard in bioinformatics research, because they perform very well even when the data are high dimensional and there are scarce data samples to train the classifier [10]. For training, SVM search for the set of support vectors that provide the greatest separation between classes, by a linear discriminant function, and hence yield results that are the most likely to be generalizable. Specifically, in its primal form, SVM tries to maximize the norm of the discrimination function weights subject to the correct classification or prediction of the desired input. In the dual formulation of the learning problem, SVM looks for the contribution of the sample data vectors to the discriminant function that minimizes the prediction error. The dual problem can be solved very efficiently by linear programming methods, though its complexity grows with the number of samples; hence SVM are not well suited for big data problems. The implementation of SVM classifiers most widely used in this field is LibSVM (http://www.csie.ntu.edu.tw/~cjlin/libsvm/) and has been shown to provide the greatest efficiencies. For the sake of complete exploration, we produce classification results with the Weka (http://www.cs.waikato.ac.nz/ml/weka/) and scikit-learn (http://scikit-learn.org/stable/) implementation of LibSVM. Variations in implementation details, that is, random number generator, may produce differences in the classifier performance.

Moreover, we try an innovative ensemble classifier building method called LibD3C [11], which is a hybrid method using *k*-means clustering on the distance matrix between classifiers, built from their distances between classification output distributions over the training sample, and a combination of dynamic selection and circulation of the selected classifiers. Initially, the process generates a large number of classifiers; that is, it creates and overparameterized system and proceeds to prune and select the best performing combination. The *k*-means step aims to select cluster representative classifiers, while the second step aims to select classifiers that are maximally diverse, as measured by the interrater agreement and the majority voting error. The dynamic selection uses sequential forward and backward selection. Individual classifiers include decision trees, SVM, and other ensembles such as Adaboost. We have used the implementation available in Weka.

It is customary in machine learning applications to estimate the performance of the classifiers for unknown data in terms of accuracy, sensitivity, and specificity through 10-fold cross-validation, which is carried out by dividing the data into 10 subsets and repeating the training and testing of the classifiers using each set in turn for the testing, while the other nine sets are pooled together for the training. The average performance obtained from these repeated training and testing experiments is an unbiased estimate of the performance that we can expect when new cases arrive.

We want to make statements about the relevance of variables and their importance for the prediction of the patient reentry. To assess the predictive power of the variables for patient readmission, Breiman's method [12] consists of calculating the mean Gini index associated with the variable when building a decision tree on the training dataset. The Gini index is a measure of the impurity of the split resulting from applying a decision at the node of a decision tree being built to classify the data. This method is implemented in the scikit-learn library.

To assess whether differences between populations were significant, we used the Welch *t*-test [3], which tests the null hypothesis that two populations have the same mean value, without assuming equal variances.

## 3. Results

We performed the processes described in the previous section to the data from each group separately: the Welch *t*-test, the importance of the variables, and the classification validation results for the classifiers introduced above.  Table 1 shows the age and sex ratio for each stratification group and sample sizes for each of the classes, so it can be appreciated that class distributions were not well balanced. This represents a major problem in the development of classifiers; nevertheless, we were able to achieve high prediction of readmissions (specificity) in all population groups.  Table 2 shows the *P* values of the *t*-tests for the most significant variables in each one of the stratification categories.  Table 3 gives the classification results of the sensitivity, specificity, and accuracy for discriminating between readmitted and nonreadmitted patients. Classifier performance distinguishing between patients who were and were not readmitted (within 30 days) in terms of average accuracy (AC), sensitivity (SN), and specificity (SP) over a 10-fold cross-validation achieved by the best classifier tested was as follows in each of the groups: 93.62% AC, 71.43% SN, and 100% SP in CM; 100% AC, 100% SN, and 100% SP in HF; 86.25% AC, 88.3% SN, and 86.3% SP in COPD; and 89.66% AC, 42.11% SN, and 98.97% SP in DM. In the comparison between the classifier training methods, we find that LibD3C does not improve SVM implementations; however, it seems to be more robust to class imbalance, as the sensitivity and specificity values are more balanced than in the SVM results. The scikit-learn version of the SVM implementation gives more optimistic results than the Weka, which may be due to differences in the way that the cross-validation is carried out. However, there is one case when this effect is inverted, so no definitive conclusion can be made. Nevertheless, all classifiers provide high classification performance, which is a positive evidence towards the development of such predictors for ED management.

**Figure 1:**
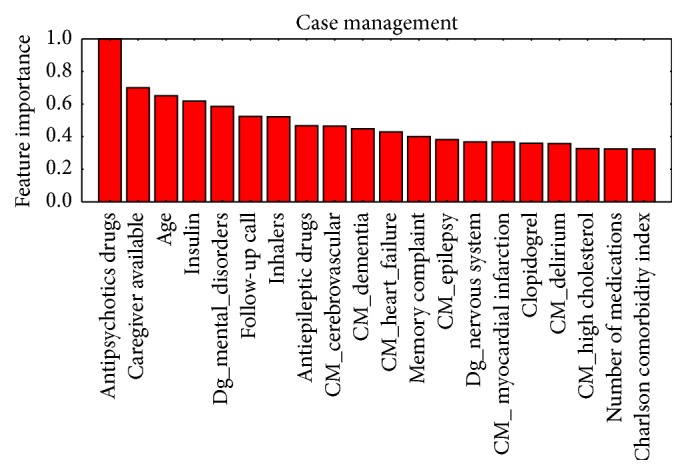
Ordered by their importance, the variables with the greatest predictive value for readmission in the case management group.

**Figure 2 fig2:**
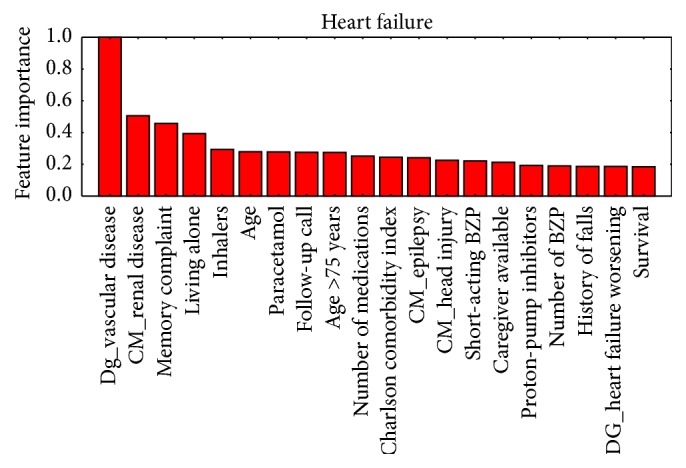
Ordered by their importance, the 20 variables with the greatest predictive value for readmission in the heart failure group.

**Figure 3 fig3:**
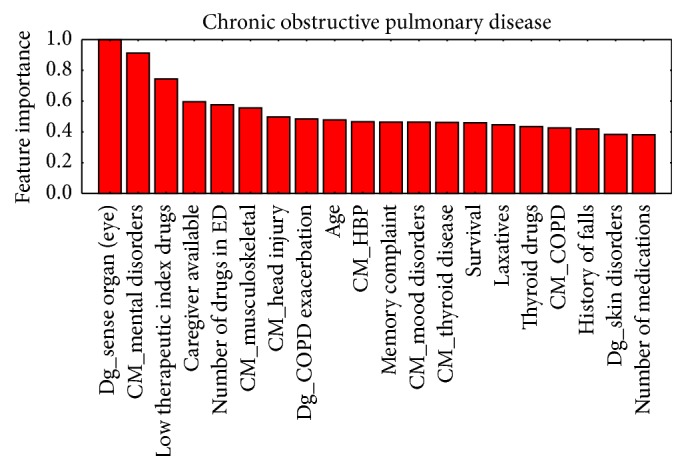
Ordered by their importance, the 20 variables with the greatest predictive value for readmission in the chronic obstructive pulmonary disease group.

**Figure 4:**
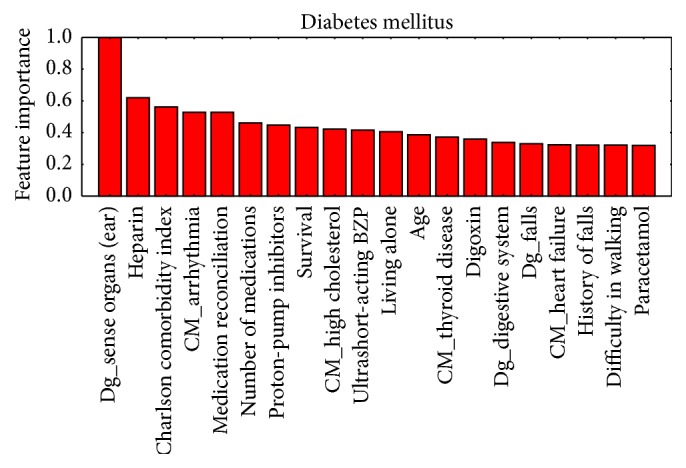
Ordered by their importance, the 20 variables with the greatest predictive value for readmission in the diabetes mellitus group.

Figures 1 [Fig fig2] [Fig fig3]–4 show, in decreasing order of importance, the 20 variables with the greatest predictive value for readmissions for each of the stratification groups (CM, HF, COPD, and DM). It is worth noting that there is a relatively small overlap between the sets of variables with significant differences and those with the greatest power for discriminating between readmitted and nonreadmitted patients. For example, in the CM group (Figure 1), only age and medication reconciliation appear in both sets. The variables with the greatest discriminating power were age, comorbidity, reasons for consultation, social factors, and drug treatments.

## 4. Discussion

Prediction of rehospitalization within a short period of time after discharge may reduce at least 10% of its costs [13]. This is especially true in elderly patients [14]. Previous long-term hospitalization is the most predictive variable [15, 16]. A time span of 30 days to evaluate undesired readmission to ED has been found to be most clinically relevant [17]; hence, we focus our study in this time frame. The studies conducted to date to identify patients with the highest risk of ED readmission have had mixed results [17–19]. The novelty of our study is that it has been carried out in a previously stratified population. We have confirmed that the establishment of such stratification groups in patients makes it possible to predict readmissions with great accuracy independently for each group. Hence, the predictors we have identified represent a useful tool for providing better care with greater efficiency by guiding clinicians as to how to focus their work to improve the care. Examining together Tables 2 and 3 and Figures 1–4, it can be appreciated that it is not necessary that variables have statistically significant differences among populations to be able to build accurate predictors or to calculate the value of each variable in the prediction of readmissions without the need to group them, allowing us to assign specific weights to, for example, each disease or drug.

The reasons why these patients attend the ED are diverse and have a role as readmission predictors. Notably, in level 2 groups (HF, DM, and COPD), patients with illnesses associated with a single organ, exacerbation/worsening or stabilisation of the primary condition plays a role in the risk of readmission, though not a dominant one.

Our findings of age and comorbidity as predictive factors for ED readmission in all four stratification groups agree with previous published results [20]. It is well known that most elderly patients have multiple concomitant health problems, many of which have a significant impact on the planning of treatment. Our study highlights the role of mental health comorbidity in the four groups analysed. Numerous studies have emphasised the importance of detecting mental illness in the ED, for the potential associated risks, and consider the assessment of mental status to be an indicator of the quality of care provided [21]. It has been estimated that a quarter of older adults seen in EDs have cognitive deterioration, while 10% have delirium and 20% depression [22, 23]. In the HF group, renal and vascular disease comorbidities emerge as reasons for ED consultation. It is common that older individuals have these diseases at the same time, and previous studies have included them as predictors of readmission in elderly patients with HF [24]. Interestingly, in the COPD group, the reasons for consultation include ophthalmological problems. A growing number of studies describe eye complications attributable to multiple factors in COPD patients [25]. Taking into consideration DM as a systemic condition, we found that disorders of the ear, nose, and throat were associated with a higher risk of readmission. It has been observed previously that complaints due to dizziness with peripheral vestibular symptoms are increasingly common in older adults, especially in those with diabetes [26]. Sensory symptoms in this age group are a problem, not only for their high prevalence, but for the greater associated risk of negative health outcomes [27].

Various authors have analysed the relationship of ED readmission and hospital admission with multiple medications and its impact on rates of morbidity and mortality [28]. In agreement with other authors [29], our results indicate that medication is an important predictor of readmission. It is estimated that 40% of over-65-year-olds take 5 to 9 different regular medications and 18% take more than 10 every day [28]. We found that readmission is influenced not only by the number of medications, but also by the type of treatment prescribed and whether there has been medication reconciliation. These findings are consistent with results of ongoing research focused on detecting potentially inappropriate medications in older adults due to over- and underprescribing [30]. In this study, we did not specifically investigate medication reconciliation. However, we found that medications such as psychotropic drugs (neuroleptics, antidepressants, and hypnotics), drugs with narrow therapeutic margin (digoxin and phenytoin), and those with a low therapeutic index had an impact on the risk of ED readmission and these are among the drugs identified in available tools for measuring potentially inappropriate prescribing in older adults [31].

Social and family factors, such as having a caregiver available or living alone, are not irrelevant to EDs and play a key role in enabling these patients to reach the hospital [29, 32]. Our study identifies these factors as one of the most important variables in the risk of 30-day readmission.

An important conclusion of this study is that the care provided in the ED for the four groups of patients studied must go beyond treating the disease, paying attention to appropriate interventions preventing readmissions [33]. To date, there has been a general trend in the EDs towards underestimating the impact on patient health outcomes of factors such as functional deterioration, psychosocial dysfunction, dementia, and caregiver burden or the lack of a caregiver [34, 35]. Our results are in line with the growing recognition of elderly individuals as a group with special needs in the ED [36]. Advances in this area may be very useful for progressing in the definition and adoption of principles of care for older patients in EDs, following the lead of paediatrics [30], avoiding efforts for the prevention of readmission mistakenly focused exclusively on specific diseases [37].

In summary, the results of this study show that population stratification allows predicting ED readmissions with high accuracy, as well as identifying the most important influencing variables, enabling specific interventions to be initiated to improve quality of care for older patients who present a growing burden on emergency services.

We plan to extend the study for a longer period of time to obtain larger sample sizes. Other limitations of the study are as follows: (a) the coding of the reasons for ED consultations is based on the main diagnosis; (b) there is lack of information regarding quality of life, level of independence, and severity of the disease; (c) the data collected on patients' functional status are actually limited to their ability to walk and personal history of falls.

## Supplementary Material

Supplementary material contains the detail enumeration of the demographic and other variables of the patients which describe their profile. Classification were performed on the basis of these variables and/or the features extracted from them.

## Figures and Tables

**Table 1:** Demographic data. Distribution per each population stratum.

	Case management	Heart failure	Chronic obstructive pulmonary disease	Diabetes mellitus
Age, years^1^	77.53 ± 10.79	84.79 ± 5.91	76.34 ± 10.02	75.84 ± 10.75
Male/female	68/31	31/50	60/25	68/58
Readmissions	22.22%	9.87%	18.82%	16.11%

^1^Values are expressed as mean and standard deviation.

**Table 2:** *P* values in the *t*-test for the most significant variables in each of the stratified groups.

Variable	*P*
Case management	
Patient age on admission	0.0054
Considered useful to make a follow-up call	0.0087
Acute myocardial infarction	0.0066
Thyroid disease	0.0013
Use of antipsychotics	0.0039
Use of inhalers	0.0034
Diagnosis of chronic obstructive pulmonary disease exacerbation	0.0021
Heart failure	
Acute myocardial infarction	0.0001
Dementia	0.0001
Number of medications prescribed on emergency department discharge	0.0000
Diagnosis of gastrointestinal illness	0.0020
Chronic obstructive pulmonary disease	
Dementia	0.0071
Depression	0.0038
Use of anticoagulants	0.0071
Genitourinary problems	0.0021
Use of opioids	0.0021
History of falls	0.0071
Diabetes mellitus	
Organic lesions	0.0006

*P* value of the difference between readmitted and nonreadmitted patients.

**Table 3:** Results of the prediction using supervised classification algorithms based on support vector machines and ensemble classifiers.

Classifier/implementation	Classification results	Case management	Heart failure	Chronic obstructive pulmonary disease	Diabetes mellitus
LibSVM scikit-learn	Accuracy %	93.62	100	83.75	89.66
	Sensitivity %	71.43	100	75.00	42.11
	Specificity %	100	100	85.94	98.97

LibSVM Weka	Accuracy %	87.23	88.57	86.25	83.620
	Sensitivity %	89.0	78.4	88.3	69.9
	Specificity %	87.2	88.6	86.3	83.6

LibD3D Weka	Accuracy %	82.97	84.28	83.75	81.89
	Sensitivity %	81.7	78.0	82.2	74.3
	Specificity %	83.0	84.3	83.8	81.9
