# Supplementary material for: Risk Factors for Emergency Department Short Time Readmission in Stratified Population
Source: Biomed Res Int. 2015 Nov 17;2015:685067. doi: 10.1155/2015/685067 (PMC4664798; doi:10.1155/2015/685067)
Supplement: Supplementary file 1 — Supplementary material contains the detail enumeration of the demographic and other variables of the patients which describe their profile. Classification were performed on the basis of these variables and/or the features extracted from them. [file 685067.f1.docx]

Supplementary material

VARIABLES

**-Sociodemographic data and baseline status:** Sex, age, and social assessment: place of residence (own home/nursing home), living alone, caregiver available, telecare service.

**-Personal history:** high blood pressure, high cholesterol, smoker, arrhythmia, fainting, head injury, acute myocardial infarction, congestive heart failure, peripheral vascular disease, cerebrovascular disease, dementia, delirium, depression, mood disorders, other psychiatric illnesses, chronic obstructive pulmonary disease, asthma, peptic ulcer disease, diabetes mellitus diabetes mellitus with organic lesions, hemiplegia , kidney disease, mild liver disease, moderate liver disease, cancer, leukaemia, lymphoma, solid tumour metastasis, AIDS, peripheral venous thrombosis, pulmonary thromboembolism, bone and joint disease, thyroid disease, epilepsy, frailty: ability to walk (difficulty to walk), history of falls, history of cognitive deterioration, memory complaints/delirium, living alone, presence/absence of caregiver, use of ≥ 5 medications, medication reconciliation in the emergency department, considered useful to carry out a follow-up call, Charlson comorbidity index, survival.

**- Reasons for consultation at the ED/Diagnoses made:** chronic obstructive pulmonary disease exacerbation (diagnosis of chronic obstructive pulmonary disease exacerbation), other reasons associated with the respiratory system (respiratory diagnosis), diagnosis of heart failure worsening, other reasons associated with the cardiovascular system (cardiovascular diagnosis), kidney and urological disease (nephro-urological diagnosis), decompensated diabetes (diagnosis of decompensated diabetes), mental illness (diagnosis of psychiatric condition), peripheral vascular disease (diagnosis of vascular disease), reasons associated with ear, nose and throat (diagnosis of disorders of the ear, nose or throat), falls (diagnosis of falls), gastrointestinal disease (diagnosis of gastrointestinal disease), neurological disease (diagnosis of neurological disease), bone and joint disease (diagnosis of bone or joint disease), skin disorders (diagnosis of skin disorders), ophthalmologic disorders (diagnosis of ophthalmologic disorders)

**-Regular medications and other treatments:** Proton-pump inhibitors, digoxine, non-steroidal anti-inflammatory drugs, low therapeutic index drugs, short-acting benzodiazepines (6-24 hours), long-acting benzodiazepines, medium-acting benzodiazepines, ultrashort-acting benzondiazepines, number of benzodiazepines, anticoagulants: aspirin, clopidogrel, heparin, oral antidiabetics, insulin, antiarrhythmic drugs, hypotensive drugs, diuretics, lipid lowering drugs, antiepileptic drugs, antiparkinson drugs, anticholinesterases, ,memantine, antidepressants, antipsychotics, inhalers, genitourinary drugs, thyroid drugs, laxatives, opioids, paracetamol, nitroglycerin, number of medications taken regularly, number of medications prescribed from emergency department (number of medications prescribed on emergency department discharge).
